# Comparison of short-stem with conventional-stem prostheses in total hip arthroplasty: an 8-year follow-up study

**DOI:** 10.1007/s00402-020-03519-y

**Published:** 2020-06-22

**Authors:** Alexander Zimmerer, Stefanie Slouka, Stefan Kinkel, Thomas Fritz, Stefan Weiss, Christian Sobau, Wolfgang Miehlke

**Affiliations:** 1 ARCUS Sportklinik Pforzheim, Rastatterstr. 17-19, 75179 Pforzheim, Germany; 2 Department of Orthopaedics, University of Greifswald, Ferdinand-Sauerbruch-Straße, 17475 Greifswald, Germany

**Keywords:** Total hip replacement, Short stem, Outcome, Hip pain, Hip prosthesis, Young patient

## Abstract

**Purpose:**

Coxarthrosis is a common disease of the adult hip joint. Elderly patients have mainly been treated with total hip arthroplasty (THA); however, younger patients are increasingly affected. Short-stem prostheses were developed for this special patient group. There have been few studies on the clinical outcomes of this type of prosthesis. This study compared the mid-term results of a short-stem prosthesis and a standard-stem prosthesis 8 years after implantation.

**Methods:**

According to our clinical registry, patients who received a short-stem prosthesis before 2011 were identified. Patients in the standard-stem prosthesis group were matched based on the sex, age, height, weight, and degree of arthrosis. At the follow-up time, the modified Harris Hip Score (mHHS), University of California Los Angeles (UCLA) activity score and visual analog scale (VAS) pain score were collected and compared with the preoperative values.

**Results:**

Fifty-five patients could be matched and analyzed for both groups. No patients needed revision surgery. In both groups, there were significant improvements at the follow-up time. The pre- and postoperative mHHSs, UCLA scores, and VAS scores were 41.9 and 95 (*p* < 0.0001), 3.75 and 7.9 (*p* < 0.0001), and 7.6 and 0.9 (*p* < 0.0001), respectively, in the short-stem group and 44.8 and 96.25 (*p* < 0.0001), 3.6 and 7.7 (*p* < 0.0001), and 7.7 and 0.9 (*p* < 0.0001), respectively, in the control group, with no significant differences between the groups at the follow-up time.

**Conclusion:**

The short-stem prosthesis provides mid-term results comparable to those of a standard-stem prosthesis. In both groups, excellent patient-reported outcomes were achieved after an average of 8 years.

**Level of evidence:**

IV.

## Background

Coxarthrosis is a common disease of the adult hip joint and is typically treated by total hip arthroplasty (THA) if conservative therapy is not successful. Thus, THA is one of the most successful orthopedic surgeries performed today [1–3]. In the past, elderly patients have mainly been treated with THA, and this is increasingly performed in younger patients, who have higher demands and expectations [4]. In the US, for example, the prevalence of coxarthrosis was estimated to be 0.83% in the total US population and to increase with age up to 5.26% in those aged 80 years [5]. However, the incidence of THA has increased for younger patients over the last decade as well [6].

In the past, young patients have been considered at a higher risk for revision due to their higher activity level relative to that of elderly patients. According to the Finnish Arthroplasty Registry, the 10-year revision rate in patients younger than 55 years who underwent THA is approximately 6% [7]. To account for this fact, special types of prostheses have been developed to preserve the bone stock for facilitating future revisions [8]. The design of these short-stem prostheses focuses on metaphyseal fixation to provide more physiological loading in the proximal femur and to reduce the risk of stress shielding [9–11]. These implants have shown excellent short-term results in primary osteoarthritis [12]. And even in the case of osteonecrosis of the femoral head revision rates did not differ from those in primary osteoarthritis [13]. However, short-stem prostheses must demonstrate an outcome equivalent to that of regular length, cementless stems to support routine use in primary THA.

One of these short-stem prostheses is the Nanos^®^ (Smith & Nephew, Marl, Germany), which has been available since 2004. This stem is made of a titanium alloy coated with calcium phosphate on approximately 75% of its surface. It is wedged in the sagittal and coronal plane with a curved distal end, providing multipoint cortical contact and loading on both the calcar region and proximal lateral cortex. Regarding the short-term radiological outcome, there have been a few studies analyzing migration over the first few years after implantation. These studies found only a slight initial migration within 3 months after implantation and stable conditions after 2 years [14, 15]. However, only 4 studies assessing the clinical and functional outcomes of Nanos implantation are available [15–18]. Overall, these studies report results after a follow-up period of 2–5.6 years. None of the mentioned studies compared the results with those of a control group, and there are no studies with a follow-up period longer than the 5.6 years mentioned.

Therefore, the present study aimed to analyze the results at 8 years after the implantation of a Nanos short-stem prosthesis and compare the results with those after the implantation of a standard-stem prosthesis in a matched patient group.

## Methods

**Fig. 1:**
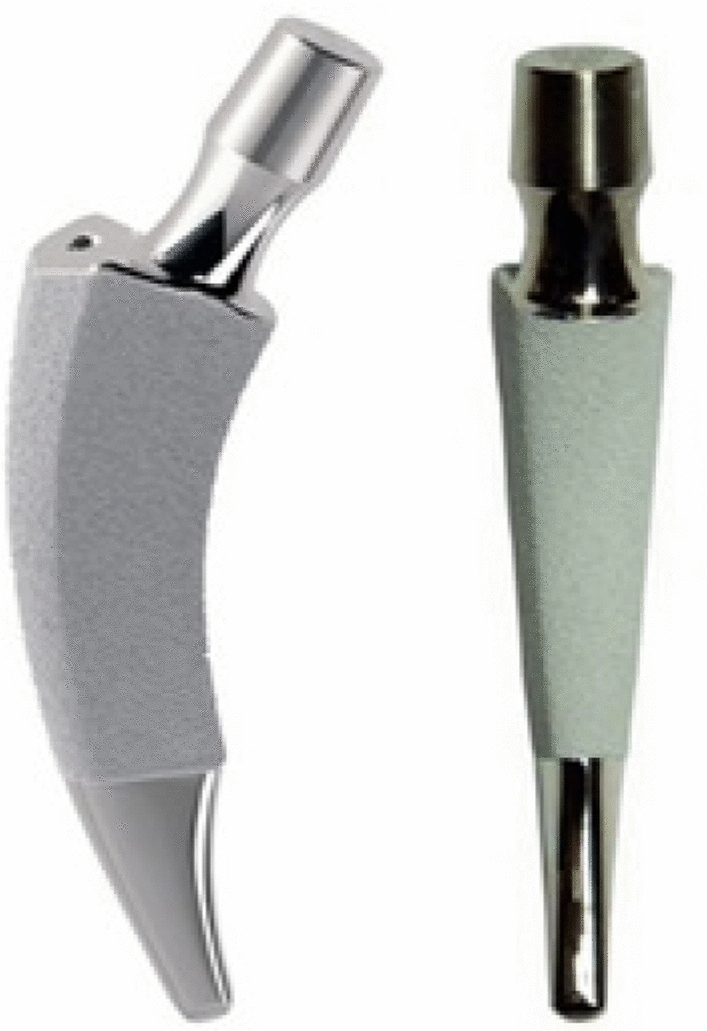
The Nanos^®^ femoral neck prosthesis (Smith & Nephew, Marl, Germany)

This was a single-center, retrospective study. After gaining the approval of the local ethics committee, we retrospectively reviewed our hospital registry to identify all patients who received a Nanos short-stem prosthesis before 2011 in our institution (Fig. 1). To create a control group of patients who received a standard-stem prosthesis (Corail^®^, DePuy, Warsaw, IN), such patients were matched by age at the time of implantation, height, weight, sex, and degree of arthrosis. In principle, patients younger than 65 years are informed about the possibility of a short-stem prosthesis (including partial weight bearing, missing long-term data, etc.), whereupon they could decide on a standard-stem or short-stem. Contraindications for the Nanos short-stem prosthesis were considered in the selection: Marked osteoporosis, marked coxa valga with a femoral neck angle > 145°, marked coxa vara with a femoral neck implant bed < 125°. These criteria were also met by the control collective.

Baseline demographics and clinical and radiological details were obtained a retrospective review of electronic medical records. The degree of arthrosis was assessed using the preoperative anteroposterior (AP)-pelvic and cross-lateral radiographs. The modified Harris Hip Score (mHHS), the University of California Los Angeles (UCLA) activity score and the visual analog scale (VAS) pain score were recorded preoperatively and at the follow-up time as primary outcome parameters. Informed consent was obtained from all individual participants included in the study.

### Surgical technique

**Fig. 2:**
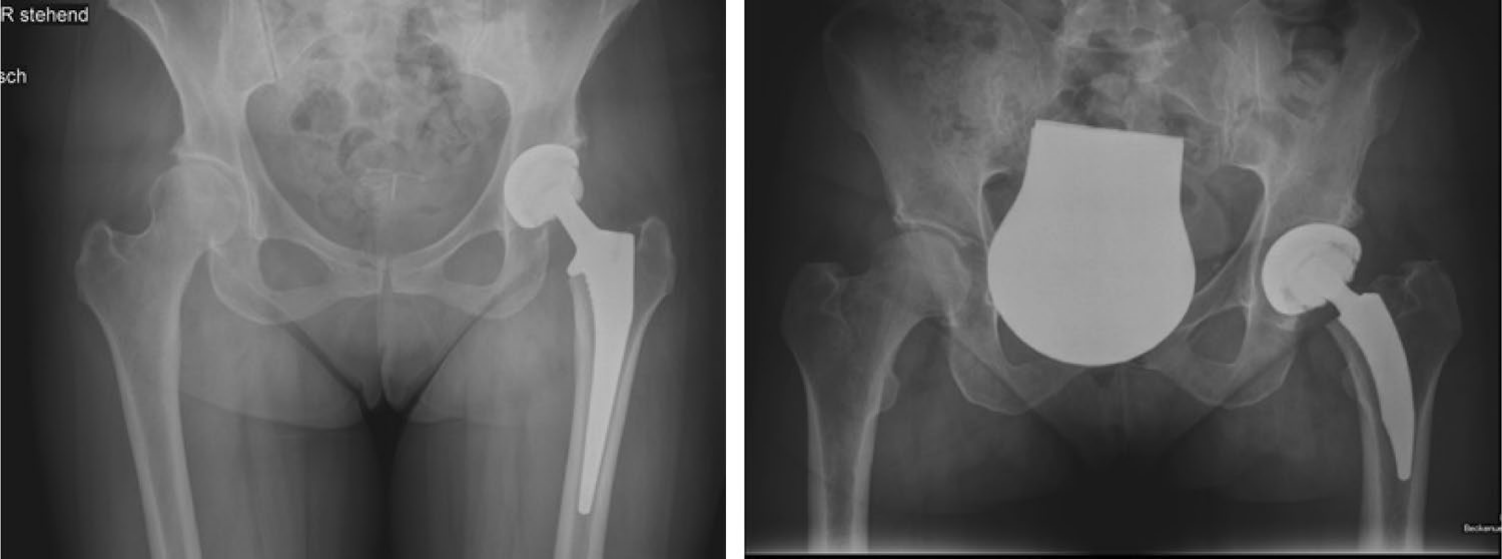
**a** Anteroposterior radiograph of the pelvis after THA with a standard stem (Corail^®^, DePuy, Warsaw, IN). **b** Anteroposterior radiograph of the pelvis after THA with a short stem (Nanos^®^, Smith & Nephew, Marl, Germany), *THA* total hip arthroplasty

All procedures were performed at our institution by 2 senior hip surgeons. The patients were operated on in the supine or lateral decubitus position under general or spinal anesthesia. A posterior or lateral approach was used, depending on the experience and preference of the surgeon, wherein one surgeon used the lateral approach, and one surgeon used the posterior approach. The indication for surgery was primary arthrosis of the hip in all patients. The prostheses were implanted cement-free according to the current technique. Figure 2 shows postoperative radiographics.

### Rehabilitation

Postoperatively, patients in the study group were allowed partial weight bearing until week 6 and then fully weight bearing thereafter. Patients in the control group were allowed full weight bearing immediately after surgery. Our local thromboprophylaxis protocol was followed in all patients, with chemical thromboprophylaxis (enoxaparin) for 4 weeks. To prevent heterotopic ossification, etoricoxib (COX-2 inhibitor) was administered at a daily dose of 90 mg for 14 days. Physical therapy started on the first postoperative day, and 6–10 days after the inpatient surgery, all patients started a rehabilitation program.

### Statistical analysis

The data were analyzed statistically using the statistical package SPSS, version 20 (SPSS version 20; IBM Corporation, USA). The results were analyzed by *t* test. Statistical significance was defined as *p* < 0.05. This study was approved by the Ethical Review Board at our institution.

This research did not receive any specific grants from commercial funding agencies or bodies.

## Results

A total of 70 patients could be matched per group. Out of these, 55 (80%) patients of the Nanos group were available at the follow-up time. Of the patients lost to follow-up, 3 died from causes unrelated to the index procedure, 10 patients could not be reached, and 2 refused to participate. The mean follow-up duration was 8 years (96 months; range 84–125 months). Baseline demographic data for both groups are presented in Table 1. Overall, no THA revisions had to be performed in any patient during the period investigated (implant survival rate, 100%). One patient in the study group had one dislocation that was treated with closed reduction and did not need any further surgical interventions. There were no cases of infection, deep vein thrombosis or other complications reported. Sixty-five percent of the short-stem prostheses and 62% of the standard prostheses were implanted via the posterior approach. All patients showed a low comorbidity with an ASA score between 1 and 2. The average degree of arthrosis was 2.4 (2–3) (*p* = 0.38) according to Tönnis in both groups.

**Table 1:** Demographics

	Females (%)	Age at THA (years)	BMI (kg/m^2^)
Study group (*n* = 55)	35	55.7 ± 8.4 (39–69)	23.7 ± 4.2 (17–32)
Control group (*n* = 55)	35	56.5 ± 8.2 (36–70)	24.8 ± 3.4 (17–31.5)
*p* value		0.514	0.315

Values are shown as the mean ± SD (range)

*THA* total hip arthroplasty, *BMI* body mass index

A significant improvement was observed in the mHHS, UCLA activity score and VAS pain score within both groups. However, no significant difference was found when comparing the two groups.

In the study group, the mHHS increased from 41.9 ± 15.2 (9–65) preoperatively to 95 ± 11.0 (50–100) at the follow-up time (*p* < 0.0001). The mHHS in the control group increased significantly from 44.8 ± 14.1 (11–64) to 96.25 ± 6.0 (52–100) (*p* < 0.0001). When comparing both groups, there was no significant difference (*p* = 0.27).

Table 2 shows the mHHS results broken down into the subgroups poor, fair, good and excellent. In both groups, over 90% of patients achieved values greater than 90 points at the follow-up time.

**Table 2:** Number of patients according to mHHS

mHHS	Poor (< 70)	Fair (70–79)	Good (80–89)	Excellent (> 90)
Study group, preoperative	55	0	0	0
Study group, follow-up	2	0	2	51
Control group, preoperative	54	1	0	0
Control group, follow-up	1	0	2	52

The UCLA activity score in the study group was 3.75 ± 2.2 (1–8) points preoperatively and significantly improved to 7.9 ± 2.0 (3–10) points postoperatively (*p* < 0.0001). In the control group, the UCLA activity score improved significantly from 3.6 ± 1.6 (1–8) to 7.7 ± 1.7 (3–10) points (*p* < 0.0001). There was no significant between the groups (*p* = 0.34).

In the study group, the VAS pain score significantly decreased from 7.6 ± 1.5 (4–10) to 0.9 ± 0.9 (0–7) points (*p* < 0.0001). A decrease from 7.7 ± 1.5 (4–10) to 0.9 ± 0.9 (0–7) points (*p* < 0.0001) was also observed in the control group. A comparison of the two groups showed no significant difference (*p* = 0.49).

## Discussion

This study evaluated the mid-term outcomes in patients younger than 65 years after the implantation of a Nanos short-stem prosthesis when compared with a standard-stem prosthesis after a mean follow-up period of 8 years. The use of this type of stem was associated with a significant improvement in the mHHS, UCLA activity score and VAS pain score. Overall, the results were comparable to those in the matched control group.

To the best of our knowledge, only 4 studies have investigated the clinical and functional outcomes after the implantation of this short-stem prosthesis. Our study has the longest follow-up period, with an average of 8 years. In addition, none of the 4 publications compared the results with those in a control group. Table 3 shows an overview of the 4 available studies.

**Table 3:** Overview of existing literature

Study	Sample size (hips)	Mean age (years)	Females (%)	F/U time (years)	Preoperative HHS	F/U HHS	*p* value
Ettinger et al. [16]	72	63	51	5.2	47.3	97.6	NR
Kaipel et al. [15]	49	64	53	2	47.9	98.1	< 0.01
Stadler et al. [17]	84	61.6	46	2.3	36.6	94.5	NR
Capone et al. [18]^a^	37	51.5	3	5.6	53	90	< 0.001

*F/U* follow-up, *NR* not recorded, *NSE* not specifically evaluated, *HHS* Harris Hip Score

^a^This study analyzed results after short-stem THA for osteonecrosis of the femoral head

In the present study, the mHHS significantly increased from 41.9 (range 9–65) to 95 (range 50–100) points at the follow-up time in the study group and from 44.8 (range 11–64) to 96.25 (range 52–100) in the control group. In both groups, 93% of the patients showed excellent results. All 4 available studies investigating the clinical outcome after the implantation of a short-stem prosthesis used the HHS. Capone et al. reported an improvement from 53 (range 35–67) to 90 (range 71–100) points after a follow-up period of 5.6 years. A total of 92% of the patients achieved excellent scores [18]. Kaipel et al. found comparable HHSs, with an increase from 47.9 to 99.1 points at the 2-year follow-up visit. In their series, 94% of the patients reported excellent results. In a study by Stadler et al., the HHS improved from 36.6 ± 14.5 to 94.5 ± 8.8 points after a follow-up period of 2.3 years [17]. Another study, by Ettinger et al., reported an improvement from 47.3 ± 12.2 to 97.6 ± 0.6 after 5.2 years [16]. Looking at these studies, we were able to confirm these excellent results after an 8-year follow-up period. Generally, excellent HHSs are found after the implantation of short-stem protheses. Schmidutz et al. reported a mean HHS of 93 ± 6.3 (range 71–100) after 2.7 years [19].

An increase in the activity level was achieved in both groups. The UCLA score was 7.9 in the study group and 7.7 in the control group at the follow-up time. Capone et al. reported a significant improvement from 2.9 (range 2–4) to 6.3 points (range 4–10) after a period of 5.6 years [18]. Further data concerning this short-stem prosthesis are not available. Schmidutz et al. found an UCLA score of 7.6 for short-stem prostheses after a follow-up period of 2.7 years, which was confirmed by our study [19]. Another study, by Malcolm et al., compared young patients under 30 with patients over 30 years of age. After a follow-up period of 6.6 years, the UCLA score was 6.5 in the former group and 6.4 in the latter (control) group. However, no distinction was made between prosthetic types in this study [20]. Albers et al. investigated the clinical outcome after the implantation of a Tri-Lock Bone Preservation Stem with a follow-up period of 5 years. The UCLA score (6 ± 2) at the last follow-up time showed significant improvement (*p* < 0.0001) from the preoperative value (4 ± 2) [21]. Looking at these studies, our series shows the best results.

In our study, the change in the VAS pain score was 6.7 points in the study group and 6.8 points in the control group, which corresponds to a clinically important difference. Brokelman et al. reported an improvement of 2.6 in the VAS pain score at rest and 4.7 during activity at an average follow-up time of 2.5 years. Patients in this study received the Charnley Elite Plus total hip prosthesis (DePuy, Warsaw, IN) or the Zweymüller hip prosthesis (Zimmer, Winterthur, Switzerland) [22]. In another study, Borkelman et al. showed a strong correlation between pain and satisfaction after THA. In particular, the VAS pain score at rest and during activity and the Western Ontario and McMaster Universities Osteoarthritis Index (WOMAC), representing pain, showed the strongest correlation with satisfaction [23]. In our study, the patients had a very low VAS pain score at the follow-up time, suggesting high satisfaction.

Our results show an implant survival rate of 100%. This is contrary to the Finnish Arthroplasty Registry, which shows a revision rate of 6% after 10 years for younger patients. If this 6% was applied to our groups, 3 prostheses would have had to be revised during the study period. One reason why there were no revisions in our study, however, is certainly the small number of cases, with 55 cases per group. In the 4 existing studies, the survival rate was 100%. In none of the studies did THA with this short-stem prosthesis have to be revised [15–18]. Looking at the results of the Australian Joint Replacement Registry, the Nanos stem showed the lowest revision rate of 1.1% of all recorded short stems after a 5-year follow-up. The average revision rate of THA using a short stem after 5 years was 3.9% [12]. In the following, the results of some other available short stems will be presented. Shin et al. compared the METHA (Aesculap, Tuttlingen, Germany) stem with a conventional-length femoral stem (BiCONTACT, Aesculap, Tuttlingen, Germany) including 50 hips in each group matched for age, sex, BMI, surgical approach, and surgeon. The authors did not find significant differences between the two groups in terms of postoperative radiographic outcomes, functional outcomes or complications [24]. Other studies reported a mean survivorship for the METHA stem of 95.9% (92–98%) with stem revision as the endpoint at a mean follow-up of 4.5 years (2.8–5.8) [25–28]. Nowak et al. presented mid-term results of the collum femoris preserving (CFP) stem (Waldemar Link, Hamburg, Germany). After a mean follow-up of 6.8 years, the overall survival rate of the the femoral component was 98%. The mean Harris hip score at follow-up was 94 points [29]. Further studies investigated the Mayo stem (Zimmer Inc., Warsaw, USA). The Mayo stem demonstrated a mean survivorship of 95.4% (92.3–100%) with aseptic loosening as the endpoint, at a mean follow-up of 4.7 years (2–7.9). The mean HHS was 91 points (85–96) at the final follow-up assessment in 592 hips [8, 30–37]. Looking at these results, it can be summarized that the NANOS stem achieves comparable clinical results in a mid-term follow-up.

Our study analyzed patients under 65 years of age. For older patients no results are published for the Nanos stem. However, recent publications that examined other short stems indicate that very good results can be achieved with this type of prosthesis even for older patients. Boller et al. examined potential differences between patients under and over 60 years who underwent a total short hip stem arthroplasty in a 24-month follow-up. The HHS improved from 53.6 ± 8.2 preoperative to 93.2 ± 9.6 in the younger cohort and for the older cohort from 57.6 ± 14.8 to 94.1 ± 7.6 after 24 months. No significant differences or any influences of osseointegration and clinical outcome of the short hip stem for both groups were detectable [38].

The limitations of our study are its retrospective and nonrandomized design. THA was performed with two different approaches, whereby the distribution within the groups was almost identical. Another limitation is that there was no clinical follow-up examination of the patients, who were interviewed only by questionnaire (by telephone or post). Patient factors, such as age, individual anatomy or bone quality, can impact the surgeon’s choice of implant. In the case of an increased valgus value, the implantation of this short-stem prosthesis represents a relative contraindication. The Nanos stem does not offer the possibility of an extra lateralized model. However, the position of the stem can be changed by the course of the osteotomy. If there are problems with the offset, it is possible to solve this with a longer head, but this may result in a lengthening of the leg. In general, points, such as obesity or osteoporosis can negatively influence the result after implantation of a short stem prosthesis. The femoral configuration can also be a contraindication for a short stem. For example, an increased fracture rate has been shown for Dorr type C femora [39, 40]. In these cases, standard stem implants are recommended. Another limitation is the attrition rate; 20% of patients were lost to follow-up. The reason for this rate is mainly due to patients who could no longer be contacted or did not report back. Unfortunately, the new addresses of these patients could not be found, so that it was not possible to contact them. However, our sample size is higher than that of half of the existing studies. Furthermore, both groups had different post-treatment protocols, which is due to the anchoring and force application of the different prosthesis types. The designs of short stems strive towards a more proximal and metaphyseal transmission of load from the implant into the surrounding bone [41, 42]. In order to reduce the risk of migration impairment of osseo-integration in the first weeks [43], partial weight-bearing was performed in the Nanos patients. In contrast, patients with a standard stem could be fully loaded immediately after surgery. The collar used improves the primary achieved stability according to vertical and rotational forces [44]. Finally, our study included only a mid-term follow-up timepoint, and most femoral prostheses have excellent results at less than 8 years. Although the mid-term results are very promising, it is not clear whether these results will be sustained over the long term.

## Conclusion

In summary, this study shows excellent results in terms of patient-reported outcome scores at an average of 8 years after the implantation of a Nanos short-stem prosthesis. The results are comparable to those of the implantation of a standard-stem prosthesis; thus, the short-stem seems to be a viable alternative for young and active patients. However, further studies need to be carried out to determine the long-term durability of this type of short-stem prosthesis.
