# Correction to: Comparison of short‑stem with conventional‑stem prostheses in total hip arthroplasty: an 8-year follow‑up study

**DOI:** 10.1007/s00402-021-03984-z

**Published:** 2021-06-22

**Authors:** Alexander Zimmerer, Stefanie Slouka, Stefan Kinkel, Thomas Fritz, Stefan Weiss, Christian Sobau, Wolfgang Miehlke

**Affiliations:** 1 ARCUS Sportklinik Pforzheim, Rastatterstr. 17‑19, 75179 Pforzheim, Germany; 2 Department of Orthopaedics, University of Greifswald, Ferdinand‑Sauerbruch‑Strase, 17475 Greifswald, Germany

## Correction to: Archives of Orthopaedic and Trauma Surgery (2020) 140:1285–1291 10.1007/s00402-020-03519-y

The article Comparison of short-stem with conventional-stem prostheses in total hip arthroplasty: an 8-year follow-up study, written by Alexander Zimmerer, Stefanie Slouka, Stefan Kinkel, Thomas Fritz, Stefan Weiss, Christian Sobau and Wolfgang Miehlke, was originally published Online First without Open Access. After publication in volume 140, issue 9, page 1285–1291 the author decided to opt for Open Choice and to make the article an Open Access publication. Therefore, the copyright of the article has been changed to © The Author(s) 2021 and this article is licensed under a Creative Commons Attribution 4.0 International License, which permits use, sharing, adaptation, distribution and reproduction in any medium or format, as long as you give appropriate credit to the original author(s) and the source, provide a link to the Creative Commons licence, and indicate if changes were made. The images or other third party material in this article are included in the article’s Creative Commons licence, unless indicated otherwise in a credit line to the material. If material is not included in the article’s Creative Commons licence and your intended use is not permitted by statutory regulation or exceeds the permitted use, you will need to obtain permission directly from the copyright holder. To view a copy of this licence, visit http://creativecommons.org/licenses/by/4.0/.

The original article has been corrected.

